# Changes in short-chain fatty acids across the glycemic spectrum: a systematic review and meta-analysis

**DOI:** 10.3389/fpubh.2026.1912172

**Published:** 2026-09-11

**Authors:** Xinyu Gao, Feifei Liao, Ziqi Cao, Wenzheng Tang, Jiahui Li, Qi Chen, Yingshuai Li

**Affiliations:** 1 Wangqi Academy, National Institute of TCM Constitution and Preventive Medicine, Beijing University of Chinese Medicine, Beijing, China; 2 Dongzhimen Hospital Affiliated to Beijing University of Chinese Medicine, Beijing, China

**Keywords:** short‑chain fatty acids, type 2 diabetes mellitus, glycemic status, dynamic changes, meta‑analysis, systematic review

## Abstract

**Objective:**

Short-chain fatty acids (SCFAs) modulate insulin sensitivity, but evidence on their association with type 2 diabetes mellitus (T2DM) is inconsistent, possibly due to dynamic changes across the glycemic spectrum from normal glucose tolerance (NGT) to established T2DM. This systematic review characterizes stage-specific variation in SCFAs to assess their potential value as metabolic correlates of T2DM.

**Methods:**

PubMed, Web of Science, Embase, and the Cochrane Library were searched up to December 2025 for observational studies on SCFAs involving participants with NGT, prediabetes (pre-DM), or established T2DM. Continuous/binary outcomes were expressed as standardized mean differences (SMDs)/odds ratios (ORs) with 95% confidence intervals (CIs). Newcastle-Ottawa Scale (NOS) and Agency for Healthcare Research and Quality (AHRQ) criteria assessed study quality. Data were pooled using fixed-effects or random-effects models according to *I^2^* heterogeneity. Subgroup analyses, sensitivity analyses, and funnel plots were performed.

**Results:**

17 studies (7,371 participants) were included. Based on 6 studies, butyrate was significantly lower in T2DM than in NGT (SMD: −0.49, 95% CI [−0.96, −0.01]), and the finding was robust in sensitivity analyses, whereas the observed increase in valerate was not. No significant differences in SCFAs were found between pre-DM and NGT except for propionate (1 study). Single-study analyses using either SMD or OR indicated lower acetate levels in T2DM than in pre-DM. Fecal, but not circulating, acetate and butyrate were significantly lower in T2DM than in NGT, with a significant sample-type subgroup difference only for acetate.

**Conclusion:**

SCFA alterations were more evident in established T2DM than in pre-DM, with reduced butyrate emerging as the most consistent finding. Acetate showed a less consistent pattern, varying across glycemic comparisons and biological matrices. These findings highlight SCFAs as key metabolic correlates of T2DM and support further prospective and interventional research on their potential nutritional and metabolic relevance.

**Systematic review registration:**

https://www.crd.york.ac.uk/prospero/. The Systematic Review Registration PROSPERO (No. CRD420251245821).

## 1 Introduction

Diabetes remains a major global public health burden, affecting approximately 589 million adults aged 20 ~ 79 years worldwide in 2024 (1). Type 2 diabetes mellitus (T2DM) accounts for over 90% of diabetes cases, and its growing prevalence is closely linked to rising rates of obesity (2, 3). Obesity may promote progression from a high-risk metabolic state to established T2DM via chronic low-grade inflammation, insulin resistance and progressive β-cell dysfunction (3, 4). Characterizing metabolic alterations across this glycemic spectrum may help evaluate their potential as early indicators of T2DM.

Short-chain fatty acids (SCFAs), major products of gut microbial fermentation of dietary fiber, modulate insulin sensitivity and glucose homeostasis through G-protein-coupled receptor signaling and regulation of inflammation and energy metabolism (2, 5–7). Experimental evidence also suggests that SCFA-related microbial changes may influence energy and lipid metabolism through the gut-brain axis (8). However, population-based findings remain inconsistent. Several studies (9–11) have reported elevated fecal SCFA levels in individuals with obesity or diabetes, whereas a prospective cohort study (12) found that higher circulating SCFA concentrations, including butyrate, were positively associated with the risk of incident T2DM. Another study (13) reported that propionate and isobutyrate increased with greater impairment of glucose metabolism. Taken together, these findings suggest that the observed SCFA profiles may vary across glycemic states and biological matrices. However, most studies assessed SCFAs at a single time point within a single glycemic state. Differences in biological matrices further complicate cross-study comparisons, limiting the characterization of stage-specific SCFA patterns across the glycemic spectrum.

This systematic review and meta-analysis characterized SCFA alterations across normal glucose tolerance (NGT), prediabetes (pre-DM), and established T2DM. We quantified differences in SCFA levels, evaluated their associations with dysglycemia risk, particularly for acetate, propionate and butyrate, and examined variation by biological matrix. We sought to determine whether SCFA alterations are detectable before established T2DM, clarify their potential relevance as metabolic correlates of T2DM, and identify priorities for future prospective and interventional research.

## 2 Methods and design

### 2.1 Search strategy

This systematic review and meta-analysis protocol was prospectively registered in PROSPERO (No. CRD420251245821), with all methods conducted in strict adherence to PRISMA guidelines (14). Comprehensive literature searches were performed across PubMed, Web of Science, Cochrane Library, and Embase up to December 2025 to identify studies on SCFAs across NGT, pre-DM, and established T2DM. Search strategies integrated database-specific controlled vocabulary and SCFAs/diabetes-related free-text terms, with full details provided in Supplementary Table 1.

All citations from the four databases were imported into EndNote 20 for screening. Two investigators (Gao XY, Liao FF) independently performed two-stage screening per pre-specified inclusion/exclusion criteria (title/abstract first, then full-text), with discrepancies resolved via consensus by a third investigator (Li YS).

### 2.2 Eligibility criteria

#### 2.2.1 Inclusion criteria

(1) Population: Human participants with NGT, pre-DM, or T2DM; (2) Exposure: Glycemic status; (3) Comparison: Studies included comparisons between patients with T2DM, pre-DM, and NGT populations; (4) Outcome: SCFA levels or their associations with dysglycemia risk; (5) Study design: Cohort studies, case–control studies, or cross-sectional studies.

#### 2.2.2 Exclusion criteria

(1) Literature including reviews, meta-analyses, conference abstracts, case reports, and letters; (2) Animal experimental studies; (3) Molecular mechanistic studies; (4) Studies from which data cannot be extracted or without core outcome indicators; (5) Non-English literature.

### 2.3 Data extraction

Two researchers independently extracted target data from eligible studies and entered the data into standardized forms. Extracted information included: study characteristics (authors, publication year, sample size, study type), participant characteristics (country of origin, sex, age, body mass index [BMI]), and primary outcome measures. All continuous data were standardized to mean ± standard deviation (SD): SD was derived from standard error (SE) via the formula SD=SE × √n, and median/IQR data were converted to mean ± SD with 95% confidence interval (CI) using the McGrath et al. (15) quantile estimation and Box-Cox methods, with all conversions completed via a validated online tool. For dichotomous outcomes, covariate-adjusted effect estimates were prioritized to minimize confounding bias.

### 2.4 Quality assessment

The Newcastle-Ottawa Scale (NOS) (16) was used to assess the quality of cohort and case–control studies. The scale includes three domains (selection, comparability, and outcome/exposure assessment) with a maximum total score of 9 stars, and studies scoring ≥ 6 stars were defined as high-quality according to the original criteria. The quality of cross-sectional studies was assessed using the criteria developed by the Agency for Healthcare Research and Quality (AHRQ) (17), which includes 11 evaluation questions, with scores of 0 ~ 3, 4 ~ 7, and 8 ~ 11 defined as low, moderate, and high quality, respectively (details in Supplementary Tables 2, 3).

### 2.5 Statistical analysis

All statistical analyses and data visualizations were conducted using Review Manager (RevMan) and R software (with appropriate packages). Pooled effect sizes were reported as standardized mean differences (SMDs) (95% CIs) for continuous outcomes, given the different units or measurement scales used across studies (see Table 1), and ORs (95% CIs) for dichotomous outcomes. Heterogeneity was assessed via the *I^2^* statistic, with fixed-effects models used for *I^2^* < 50% and random-effects models for *I^2^* ≥ 50%. Sensitivity analysis was conducted by sequentially excluding individual included studies to verify the stability of the results. A funnel plot was used to qualitatively assess publication bias. In addition, subgroup analyses were performed based on sample type (fecal, circulating [plasma or serum]) and detection method (chromatography, other). Statistical significance was defined as a two-sided *P* < 0.05.

**Table 1:** Characteristics of included studies.

References	Country	Study type	Population (male/female)	Grouping criteria	Age (years) (Mean ± SD)/Median (IQR)	BMI (kg/m^2^) (Mean ± SD)	Methods for SCFA measurement	Biological matrix	Unit/Measure-ment scale	Confounders adjusted
Akanji et al. (18)	United Kingdom	Baseline of intervention studies	N = 22 (12/10) Non-diabetic = 13 (6/7) Diabetic = 9 (6/3)	NIDDM, FPG > 10 mmol/L, insulin-naïve	Non-diabetic: 41.8 ± 12.62 Diabetic: 52.1 ± 10.8	Non-diabetic: 27.0 ± 4.33 (obese subgroup: 30.6 ± 1.85; normal-weight subgroup: 22.8 ± 1.96) Diabetic: 29.5 ± 6.9 (obese subgroup: 32.3 ± 6.61; normal-weight subgroup: 23.7 ± 2.60)	Enzymatic method	Plasma	mmol/L	NR
Akanji et al. (19)	United Kingdom (Oxford)	Study 1: cross-sectional study	Study 1: N = 191 (124/67) NC = 77 (56/21) DM = 114 (68/46)	All with established diabetes	NC: 31.8 ± 10.8 DM: 56.8 ± 12.3 (NIDDM 59.0 ± 10.4, IDDM 43.7 ± 14.8)	Non-diabetic (ivGTT): 22.2 ± 2.4 Diabetic (ivGTT): 26.8 ± 4.3 Non-diabetic (OGTT): 23.5 ± 2.4	Enzymatic method	Plasma	mmol/L	NR
Wolever et al. (20)	Canada (Toronto/Montreal)	Cross-sectional study	N = 40 (25/15) NC = 18 (10/8) YNOR = 10 (6/4) MNOR = 8 (4/4) IGT = 22 (15/7)	YNOR: No OGTT screening; MNOR: 2-h OGTT glucose < 7.8 mmol/L IGT: 2-h OGTT glucose ≥ 7.8 and ≤ 11.1 mmol/L (WHO criteria)	YNOR: 30 ± 6.32 MNOR: 45 ± 8.49 IGT: 56 ± 9.38	YNOR: 24.2 ± 3.16 MNOR: 29.8 ± 5.09 IGT: 31.2 ± 4.22	Gas chromatography	Serum	μmol/L	NR
Liu et al. (21)	China (Beijing)	Cross-sectional study	N = 30 (13/17) HC = 15 (7/8) T2DM = 15 (6/9)	ADA 2005 criteria	HC: 65.3 ± 6.6 T2DM: 63.9 ± 7.3	HC: 25.63 ± 2.04 T2DM: 24.98 ± 2.93	600 MHz ^1^H NMR, CPMG pulse sequence, metabolomics analysis (untargeted)	Plasma	Normalized relative integral (×10^−2^)	NR
Adachi et al. (22)	Japan	Case–control study	N = 118 (68/50) T2DM = 59 (34/25) Control = 59 (34/25)	T2DM per JDS criteria; HbA1c 6.2–7.9% at screening	T2DM: 64.0 (57.5–69.0) Control: 62.0 (59.0–69.0)	T2DM: 23.0 (20.4–25.6) Control: 22.7 (20.8–24.3)	High-performance liquid chromatography (HPLC)	Feces	mg/g	NR
Morais et al. (23)	Portugal	Observational study (cross-sectional, ex vivo metabolomic analysis)	N = 9 (3/6) Non-Ob = 4 (2/2) Ob + Pre-T2D = 5 (1/4)	Non-Ob: HbA1c < 5.6% Ob + Pre-T2D: HbA1c 5.6–6.4%	Non-Ob: 48 ± 14 Ob + Pre-T2D: 50 ± 6.71	Non-Ob: 26.1 ± 2.0 Ob + Pre-T2D: 44.0 ± 6.26	^1^H-NMR	Visceral adipose tissue culture medium	nmol/mg wet VAT	NR
Zhong et al. (24)	China	Observational study (cross-sectional)	N = 60(34/26) NC = 30 (15/15) DM = 30 (19/11) (one sample was excluded)	ADA 2017	NC: 51.93 ± 8.62 DM: 59.10 ± 8.45	NC: 23.51 ± 2.33 DM: 24.70 ± 5.96	Gas chromatography–mass spectrometry (GC–MS)	Feces and serum	Fecal SCFA: μg/g Serum SCFA: μmol/L	NR
Salamone et al. (25)	Italy	Cross-sectional study, based on baseline data from two intervention studies	N = 71 (39/32) T2D = 43 (25/18) Overweight/obese = 28 (14/14)	Not specified; well-controlled T2D (HbA1c ≤ 7.5%) with stable diet or oral antidiabetic drugs, no diabetic complications	T2D: 63.3 ± 6.2 Overweight/obese: 43.3 ± 12.7	T2D: 31.1 ± 3.6 Overweight/obese: 29.1 ± 2.9	Gas chromatography-flame ionization detector (GC-FID)	Serum	μmol/L	NR
Smith et al. (26)	United States	Cross-sectional comparison (based on baseline data from clinical trials)	N = 29 (14/15) IR-group = 20 (11/9) R-group = 9 (3/6)	Met the criteria for prediabetes with insulin resistance (as defined by the study)	IR-group: 34.8 ± 6.26 R-group: 30.4 ± 5.1	IR-group: 28.2 ± 3.58 R-group: 23.4 ± 3.0	Gas chromatography-flame ionization detector (GC-FID)	Feces	μmol/g	NR
Li et al. (27)	China	Case–control study (based on the rural cohort of Henan)	N = 100 (37/63) T2DM = 50 (17/33) Control = 50 (20/30)	ADA criteria (FPG ≥ 7.0 mmol/L, HbA1c ≥ 6.5%, or self-reported diagnosis/antidiabetic medication use)	T2DM: 58.60 ± 7.23 Control: 59.84 ± 8.49	T2DM: 25.82 ± 3.70 Control: 23.51 ± 2.83	Gas chromatography–mass spectrometry (GC–MS)	Feces	μg/g	Age, WC, TAG, sex, education level, income, smoking, alcohol consumption, physical activity, BMI, total energy intake, cholesterol, protein
Liu et al. (28)	China	Observational study (cross-sectional; metabolomic analysis combined with OGTT)	N = 113 NGT = 30 (2/28) IGR (IFG and IGT) = 40 (11/29) NDM = 43 (19/24)	NGT: FPG < 6.1 mmol/L, 2 h-OGTT <7.8 mmol/L IFG: 6.1 mmol/L < FPG < 7.0 mmol/L, 2 h-OGTT <7.8 mmol/L IGT: FPG < 7.0 mmol/L, 7.8 mmol/L ≤ 2 h-OGTT <11.11 mmol/L NDM: FPG ≥ 7.0 mmol/L or 2 h-OGTT ≥11.11 mmol/L (WHO, 1999)	NGT: 35.5 (28.0–44.0) IGR: 39.0 (32.3–51.3) NDM: 52.0 (43.0–65.0)	NGT: 25.8 (21.6–28.1) IGR: 27.9 (24.8–32.4) NDM: 26.6 (24.2–28.4)	Nuclear Magnetic Resonance Spectroscopy (NMR)	Serum	mmol/L; % change/OR reported	Sex, BMI, fasting insulin, heart rate, smoking status, BP
Shinoda et al. (29)	Mongolia (Ulaanbaatar and Bulgan Province)	Observational study (cross-sectional comparison)	N = 66 (28/38) DO = 31 (18/13) NDO = 35 (10/25)	PDO (prediabetic obese) defined as HbA1c > 5.7% in NDO group; PDO SCFA data not reported DO: HbA1c ≥ 6.5% (NGSP criteria)	DO: 53.0 ± 7.9 NDO: 49.0 ± 7.7	DO: 33.7 ± 4.7 NDO: 32.0 ± 2.7	^1^H-NMR spectroscopy (400 MHz)	Feces	μmol/g	Age, BMI
Yang et al. (30)	China	Case–control study (based on the rural cohort of Henan)	N = 100 (37/63) T2DM = 26 Control = 74	ADA & WHO criteria (self-reported diagnosis/antidiabetic medication, FPG ≥ 7.0 mmol/L, or HbA1c ≥ 6.5%)	59.2 ± 7.87	24.7 ± 3.47	Gas chromatography–mass spectrometry (GC–MS)	Feces	μg/g	Age, sex, smoking history, alcohol drinking history, physical activity, dietary fiber intake, total energy intake, high-fat diet, BMI
Zhang et al. (31)	China	Cross-sectional analysis using baseline data from an intervention trial	N = 20 (9/11) Healthy controls = 8 (4/4) obese T2D = 12 (5/7)	Healthy controls: no metabolic diseases, BMI < 24 kg/m^2^, and normal FPG and OGTT Obese T2D: diagnosed according to Chinese Guidelines for the Prevention and Treatment of Type 2 Diabetes (2020 Edition), with BMI ≥ 28 kg/m^2^ (Asia-Pacific obesity criteria)	Controls: 33.00 ± 6.02 T2D: 36.25 ± 9.29	Controls: 20.89 ± 1.50 T2D: 36.67 ± 5.94	Liquid chromatography–tandem mass spectrometry (LC–MS/MS)	Plasma	ng/mL	NR
Llauradó et al. (13)	Spain	The prospective cohort study Di@bet.es	Baseline population: 5,076 SCFA analysis included: 2,900 participants (659 baseline T2D patients, 2,241 non-T2D individuals)	Prediabetes: IFG (FPG 6.1–6.9 mmol/L) or IGT (2 h-OGTT 7.8–11.0 mmol/L) T2D: ADA criteria (FPG ≥ 7.0 mmol/L, 2 h-OGTT ≥ 11.1 mmol/L, HbA1c ≥ 6.5%, or glucose-lowering medication)	Non-T2D: 47.2 ± 14.8 T2D: 56.0 ± 12.1	Non-T2D: 27.3 ± 4.6 T2D: 31.1 ± 5.0	Gas chromatography–mass spectrometry (GC–MS)	Plasma	μmol/L	Age, sex, family history, impaired glucose tolerance, obesity, hypertension, dyslipidemia
Puig et al. (32)	Spain	Cross-sectional study (substudy based on the Di@bet.es cohort)	N = 88 (52/36) NonDM = 39 (20/19) PreDM = 24 (13/11) T2D = 25 (19/6)	NonDM: FPG < 100 mg/dL and 2 h-OGTT < 140 mg/dL PreDM: FPG 100–125 mg/dL or 2 h-OGTT 140-199 mg/dL T2D: FPG ≥ 126 mg/dL, or 2 h-OGTT ≥200 mg/dL, HbA1c ≥ 6.5%, or glucose-lowering medication (ADA criteria)	NonDM: 60.1 ± 13.8 PreDM: 67.4 ± 8.2 T2D: 70.5 ± 9.4	NonDM: 27.4 ± 3.7 preDM: 29.2 ± 4.5 T2D: 29.5 ± 4.2	Gas chromatography-flame ionization detector (GC-FID)	Feces	mmol/kg (μmol/g)	WC, sex, age, TAG
Wang et al. (12)	China	Nested case–control study (based on the prospective cohort 4C study)	N = 3,414 (1,386/2,028) New-onset T2DM = 1,707 (693/1,014) Normal glucose control = 1,707 (693/1,014)	Control: FPG < 126 mg/dL and 2 h-PG < 200 mg/dL, no diabetes history or medication New-onset T2DM: FPG ≥ 126 mg/dL or 2 h-PG ≥ 200 mg/dL, or self-reported diabetes/glucose-lowering drugs	Overall: 57.56 ± 8.87 (no age difference between matched cases and controls)	NR	Gas chromatography–mass spectrometry (GC–MS)	Serum	μmol/L	Age, sex, BMI, smoking status, alcohol drinking, physical activity, SBP, HDL-C, LDL-C, TAG, AST, ALT, FPG

SD, standard deviation; IQR, interquartile range; NC, normal control; HC, healthy control; YNOR, young normal group; MNOR, middle-aged normal group; DM, diabetes mellitus; T2DM, type 2 diabetes mellitus; T2D, type 2 diabetes; Ob, obese; NGT, normal glucose tolerance; NDM/NonDM, non-diabetic mellitus; Pre-T2D/PreDM, prediabetes; IR-group, insulin resistance group; R-group, reference group; IGR, impaired glucose regulation; IFG, impaired fasting glucose; IGT, impaired glucose tolerance; DO, diabetic obese; NDO, non-diabetic obese; PDO, prediabetes obesity; NIDDM, non-insulin-dependent diabetes mellitus; IDDM, insulin-dependent diabetes mellitus; FPG, fasting plasma glucose; OGTT, oral glucose tolerance test; HbA1c, glycated hemoglobin A1c; BMI, body mass index; PG, plasma glucose; ivGTT, intravenous glucose tolerance test; WHO, World Health Organization; ADA, American Diabetes Association; JDS, Japanese Diabetes Society; NGSP, National Glycohemoglobin Standardization Program; MHz, megahertz; CPMG, Carr–Purcell–Meiboom–Gill sequence; WC, waist circumference; TAG, triacylglycerol; BP, blood pressure; SBP, systolic blood pressure; HDL-C, high-density lipoprotein cholesterol; LDL-C, low-density lipoprotein cholesterol; AST, aspartate aminotransferase; ALT, alanine aminotransferase; NR, not reported.

## 3 Results

### 3.1 Literature search and study selection

**Figure 1:**
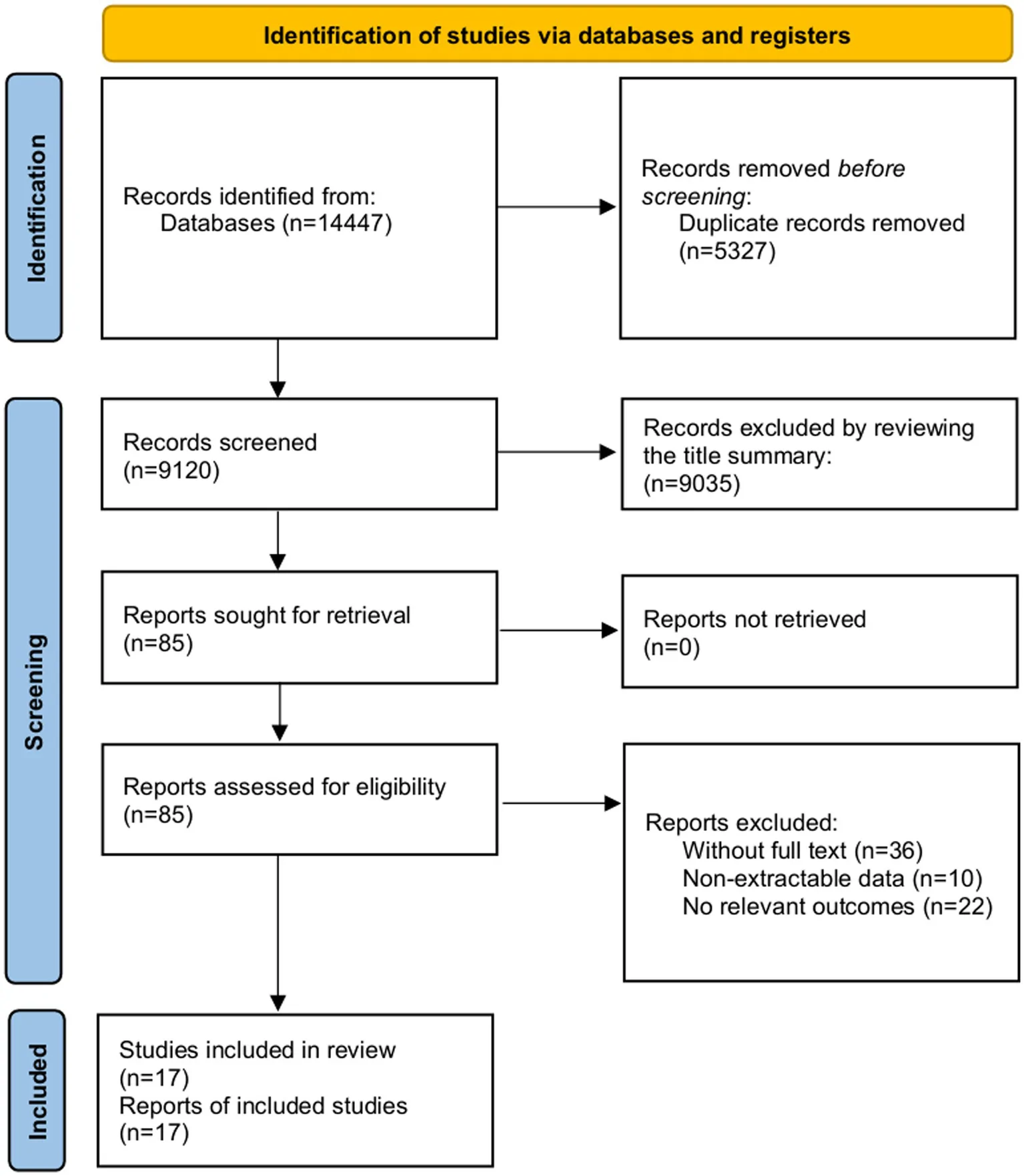
PRISMA flow diagram of identified and screened references. PRISMA, Preferred Reporting Items for Systematic Reviews and Meta-Analyses.

The initial literature search retrieved 14,447 citations across PubMed, Web of Science, Cochrane Library, and Embase. All records were imported into EndNote 20 for deduplication, leaving 9,120 unique records for title/abstract screening. 9,035 records irrelevant to SCFAs or the glycemic spectrum were excluded, with 85 potentially eligible studies remaining. 66 records were excluded in the second-stage full-text screening for ineligible study design or unextractable data, with 17 studies meeting all inclusion criteria finally included. The screening workflow is summarized in Figure 1.

### 3.2 Study characteristics

Overall, 17 eligible studies (12 cross-sectional, 1 cohort, 4 case–control; published 1988 ~ 2025) were included, enrolling 7,371 participants aged 21 ~ 80 years. Most studies classified glucose tolerance status according to American Diabetes Association (ADA)/World Health Organization (WHO) criteria, reported multivariable-adjusted ORs for outcomes, and defined overweight/obesity by BMI. BMI was explored as a potential effect modifier in our analyses. Baseline characteristics of included studies are detailed in Table 1.

### 3.3 Quality of evidence

Methodological quality was evaluated via the NOS for cohort/case–control studies and the AHRQ checklist for cross-sectional studies (details in Supplementary Tables 2, 3). Of the 5 NOS-assessed studies, 4 were high quality and 1 moderate; of the 12 AHRQ-assessed cross-sectional studies, 9 were high quality and the remaining 3 moderate.

### 3.4 Associations between SCFAs and the glycemic spectrum

**Figure 2:**
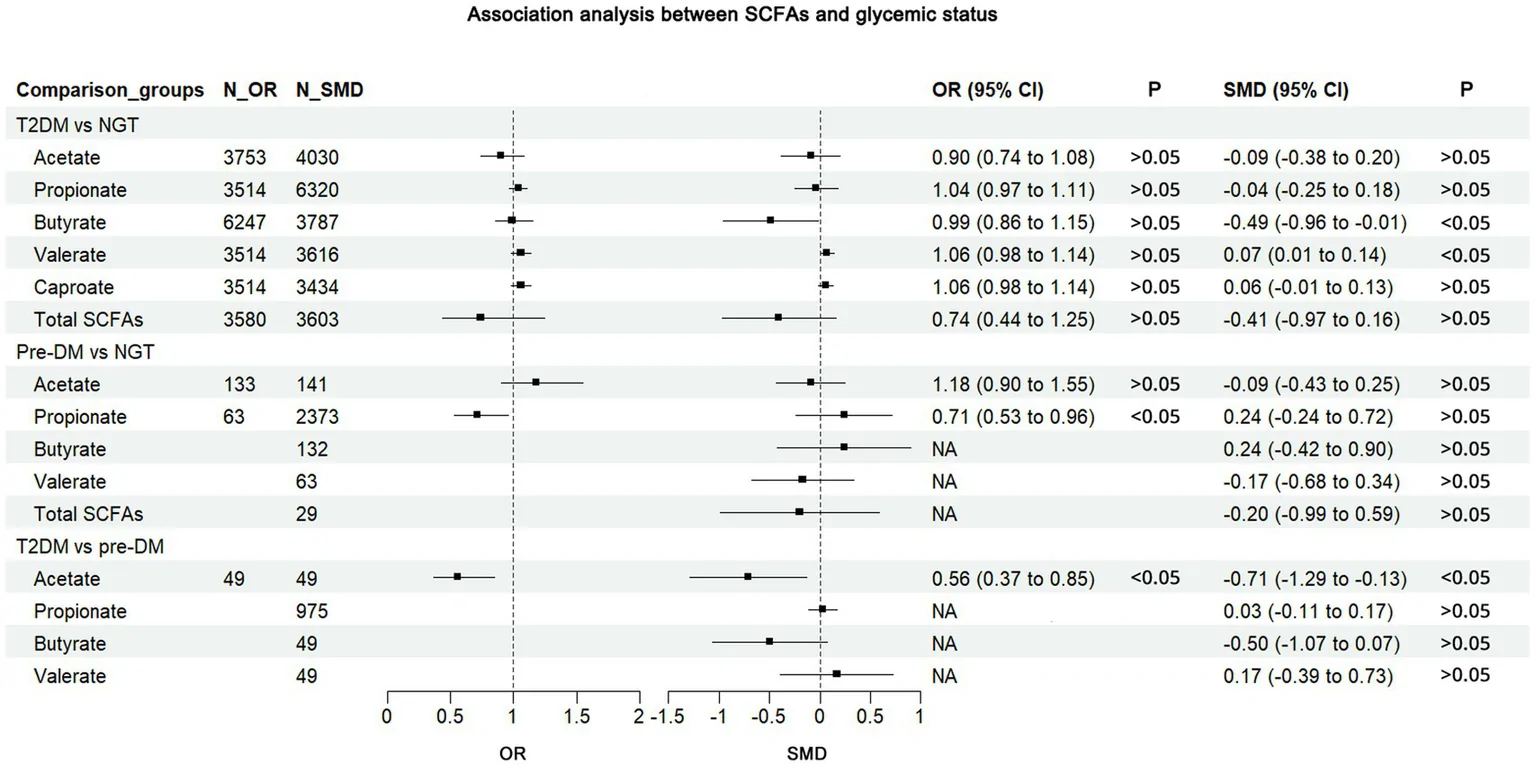
Association analysis between SCFAs and glycemic status. Effect sizes include OR (95% CI) for dysglycemia risk and SMD (95% CI) for SCFA levels. *P* < 0.05 denotes statistical significance. N_OR, sample size for OR analyses; N_SMD, sample size for SMD analyses; NA, not applicable.

In this study, we performed three independent meta-analyses to systematically evaluate the associations between SCFAs and glycemic status across the spectrum from NGT to pre-DM, and established T2DM (Figure 2; Supplementary Table 4).

#### 3.4.1 T2DM vs. NGT comparison

Pooled analyses of individual SCFA indicators showed divergent changes in their levels between T2DM and NGT populations.

For continuous outcomes, pooled analyses of SCFA indicators are summarized below. Butyrate was significantly lower in the T2DM group vs. the NGT group (SMD = −0.49, 95% CI − 0.96 to −0.01, *P* < 0.05), with extreme between-study heterogeneity (*I^2^* = 90%, *P* < 0.00001) (Figure 2; Supplementary Figure 1A; Supplementary Table 4); Valerate was significantly higher in the T2DM group (SMD = 0.07, 95% CI 0.01 ~ 0.14, *P* < 0.05), with no heterogeneity (*I^2^* = 0%, *P* = 0.66) (Figure 2; Supplementary Figure 1B; Supplementary Table 4); Acetate, propionate, and total SCFAs showed non-significant downward trends in the T2DM population (all *P* > 0.05); Caproate showed a non-significant upward trend (*P* > 0.05). Full non-significant results are presented in Supplementary Figure 2.

For the binary outcome of diabetes risk, no significant association was found between acetate, butyrate, total SCFAs and diabetes risk (all *P* > 0.05) (Figure 2; Supplementary Figure 3; Supplementary Table 4). Analyses of propionate, valerate and caproate were each limited to 2 studies, with pooled estimates driven by a single large-sample study; all three showed non-significant associations with diabetes risk (pooled ORs ≈ 1, 95% CIs crossed the null, all *P* > 0.05) (Figure 2; Supplementary Figure 4; Supplementary Table 4).

#### 3.4.2 Pre-DM vs. NGT comparison

Pooled analyses comparing pre-DM and NGT groups showed no significant between-group difference in acetate levels (SMD = −0.09, 95% CI − 0.43 to 0.25, *P* > 0.05, *I^2^* = 17%, *P* = 0.31). Propionate and butyrate showed non-significant upward trends in the pre-DM group (all *P* > 0.05) (Figure 2; Supplementary Figure 5; Supplementary Table 4). Valerate and total SCFAs were only assessed in one study each, with non-significant minimal between-group differences (Figure 2; Supplementary Table 4).

For the binary outcome of pre-DM risk, acetate showed no significant association (pooled OR = 1.18, *P* > 0.05) (Figure 2; Supplementary Figure 6; Supplementary Table 4). Only one study evaluated propionate, reporting a significant negative association with pre-DM risk (OR = 0.71, 95% CI 0.53 ~ 0.96, *P* < 0.05), though this finding has limited evidential strength (Figure 2; Supplementary Table 4).

Overall, no consistent significant differences in SCFA levels were observed between pre-DM and NGT groups, indicating that SCFA metabolic dysregulation may not be a hallmark of early-stage diabetes.

#### 3.4.3 T2DM vs. pre-DM comparison

To assess dynamic changes in SCFA levels across glycemic states, we compared SCFA levels between T2DM and pre-DM groups. In one study, acetate was significantly lower in T2DM than in pre-DM, and was associated with lower diabetes risk (Figure 2; Supplementary Table 4). Propionate showed a non-significant pooled effect (SMD = 0.03, 95% CI − 0.11 to 0.17) with inconsistent between-study effects (Figure 2; Supplementary Figure 7; Supplementary Table 4). Butyrate and valerate were only assessed in one study each, with non-significant between-group differences (Figure 2; Supplementary Table 4). Overall, only acetate showed a downward trend with increasing glycemic impairment, with no consistent and significant dynamic changes for other SCFAs.

### 3.5 Sensitivity analysis

In this study, the threshold for significant between-study heterogeneity was predefined as *I^2^* > 70%, and leave-one-out sensitivity analysis was performed to assess the robustness of pooled effect estimates and identify sources of heterogeneity.

For T2DM vs. NGT comparisons, acetate and propionate showed robust results, with pooled effect estimates remaining stable after sequential exclusion of individual studies. For butyrate and total SCFAs, excluding two outlying studies (12, 25) reduced the overall between-study heterogeneity from 90 to 56 and 35%, respectively. These two studies were the primary sources of heterogeneity, and the pooled effect sizes remained stable after exclusion. For valerate (*I^2^* = 0%), the pooled estimate was no longer statistically significant after omitting one individual study (12), suggesting the finding was dependent on a single study; cautious extrapolation is therefore recommended.

For the binary outcome of diabetes risk, the pooled estimate of acetate was sensitive to two specific studies (12, 28). Exclusion of these studies rendered the pooled effect statistically significant, indicating low robustness of the original finding. Butyrate yielded stable pooled results; removing one outlying study (30) reduced heterogeneity from 77 to 65%, with no material change in the pooled effect size. For total SCFAs, removing one influential study (12) decreased heterogeneity from 84 to 33%. After model adjustment, total SCFAs showed a significant negative association with diabetes risk (*P* < 0.05), suggesting that the overall results were susceptible to both model specification and individual study effects.

For pre-DM vs. NGT comparisons, after excluding one study (20), heterogeneity for propionate and butyrate was markedly reduced from 75 to 25% and from 68 to 0%, respectively. A fixed-effects model for propionate showed a slight elevation in the pre-DM group (*P* < 0.05), while butyrate remained statistically non-significant; this study was identified as the main source of heterogeneity. Acetate (*I^2^* = 17%, low heterogeneity) showed robust results with no change in effect estimate after any single study exclusion.

### 3.6 Subgroup analysis

**Figure 3:**
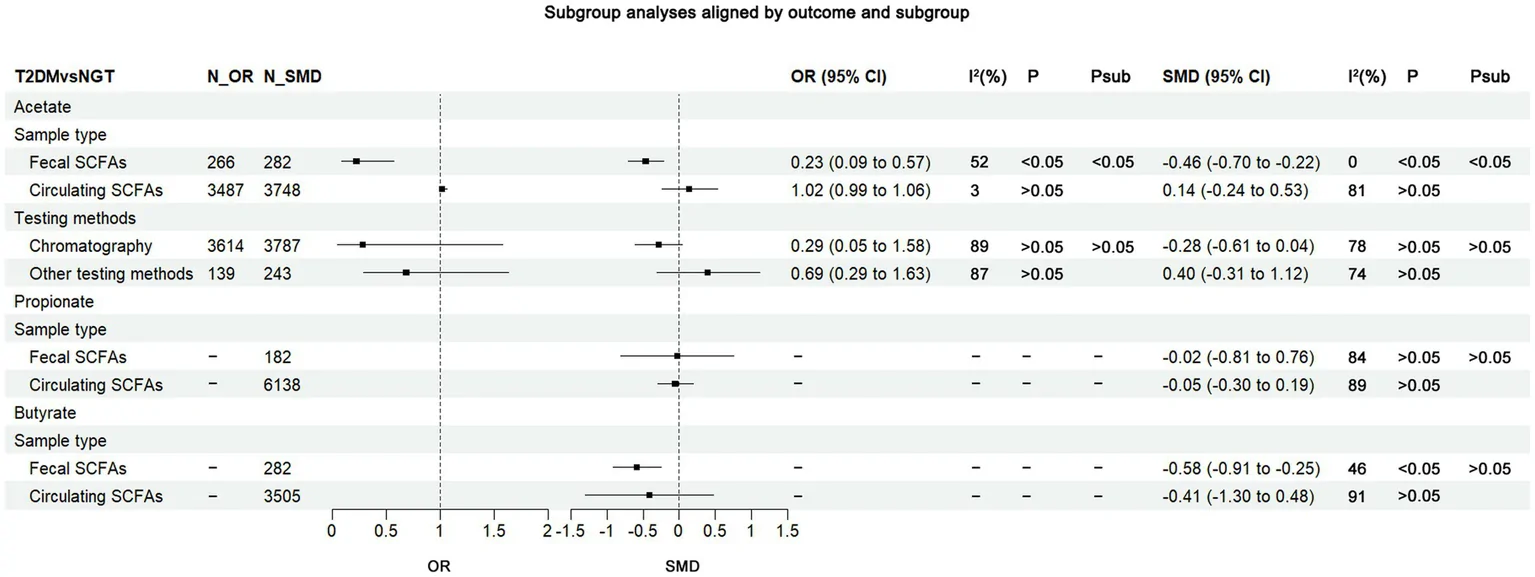
Subgroup analyses of SCFAs in T2DM versus NGT by sample type and detection method. Effect sizes are presented as OR (95% CI) for T2DM risk and SMD (95% CI) for SCFA levels. *P*sub indicates the *P*-value for between-subgroup differences. *P* < 0.05 denotes statistical significance. N_OR, sample size for OR analyses; N_SMD, sample size for SMD analyses; *I^2^*, between-study heterogeneity; −, not applicable.

Subgroup analyses were conducted according to sample type (fecal/circulating) and detection method (chromatography/other). In the subgroup stratified by sample type, fecal acetate (SMD = −0.46, 95% CI − 0.70 to −0.22, *P* < 0.05, *I^2^* = 0%) and butyrate (SMD = −0.58, 95% CI − 0.91 to −0.25, *P* < 0.05, *I^2^* = 46%) were significantly lower in the T2DM group relative to the NGT group. Fecal acetate also negatively associated with T2DM risk (OR = 0.23, 95% CI 0.09 ~ 0.57, *P* < 0.05, *I^2^* = 52%). By contrast, no significant differences were observed for these SCFAs in circulating samples (all *P* > 0.05) (Figure 3; Supplementary Figures 8, 11; Supplementary Table 5). Propionate exhibited no significant intergroup differences in either sample subgroup, consistent with the overall pooled findings (all *P* > 0.05) (Figure 3; Supplementary Figure 10; Supplementary Table 5). Stratification by detection method was not applicable to propionate and butyrate due to limited included studies, no significant intergroup differences were observed for other analyzable SCFAs (all *P* > 0.05) (Figure 3; Supplementary Figure 9; Supplementary Table 5).

### 3.7 Publication bias

The funnel plot for representative metabolites showed mild asymmetry, but quantitative publication bias tests (Egger’s and Begg’s tests) were not feasible due to sample size limitations. Publication bias could not be fully assessed, and thus the robustness of the present findings requires validation in larger-scale future studies (Supplementary Figures 12–14).

## 4 Discussion

### 4.1 Key findings

As key gut microbial metabolites, SCFAs are essential for intestinal homeostasis and systemic metabolism, with their levels modulated by dietary fiber (33). Elevated SCFAs exert anti-obesity and anti-diabetic effects (34, 35). Notably, Puig et al. (32) identified fecal acetate as a positive predictor of pre-DM but a negative predictor of T2DM, indicating a stage-specific transition from compensatory elevation to depletion in the SCFA-glycemia axis.

This systematic review and meta-analysis of 17 observational studies involving 7,371 participants evaluated SCFA alterations across the glycemic spectrum. The main findings were threefold. First, SCFA profiles did not show consistent alterations at the pre-DM stage compared with NGT, whereas acetate appeared to decline during progression from pre-DM to T2DM, although evidence for this transition was limited. Second, butyrate was significantly reduced in established T2DM compared with NGT and remained stable in sensitivity analysis, representing the most consistent SCFA alteration identified. Third, subgroup analyses suggested that sample type may partly explain heterogeneity, with fecal acetate and butyrate showing more evident reductions in T2DM than circulating SCFAs. These findings indicate that SCFA alterations in dysglycemia are unlikely to follow a uniform pattern but rather vary according to glycemic states, SCFA type, and biological specimen.

### 4.2 Compared with previous studies

Previous findings on SCFAs and diabetes remain highly inconsistent. Some small-scale studies (20, 32) have reported that individual SCFAs (e.g., butyrate and acetate) are significantly altered as early as pre-DM, potentially serving as predictive indicators for early-stage diabetes. Moreover, most previous studies have been restricted to a single glycemic state and have focused on only one or a few SCFA species, lacking a comprehensive evaluation.

In the present study, multi-stage data were integrated and a comprehensive panel of SCFAs was systematically analyzed. Our meta-analysis demonstrated that SCFA levels were largely unaltered in pre-DM. Only one study on propionate (32) suggested a potential protective association with early glucose dysregulation; however, evidence from a single study is insufficient to draw firm conclusions. The more evident SCFA alterations observed in established T2DM may indicate that SCFA dysregulation becomes more pronounced with worsening glycemic status. However, given the observational nature of the included studies, it remains unclear whether SCFA alterations precede or follow the development of T2DM. Acetate exhibited dynamic changes with deteriorating glucose metabolism, indicating that it may be a sensitive SCFA indicator of differences in glycemic states. In contrast, a significant reduction in butyrate (*P* < 0.05) was detected in patients with overt diabetes. Although these findings are consistent with previous studies, they should be considered preliminary evidence and require further validation in prospective longitudinal studies.

### 4.3 Biological mechanisms of butyrate

**Figure 4:**
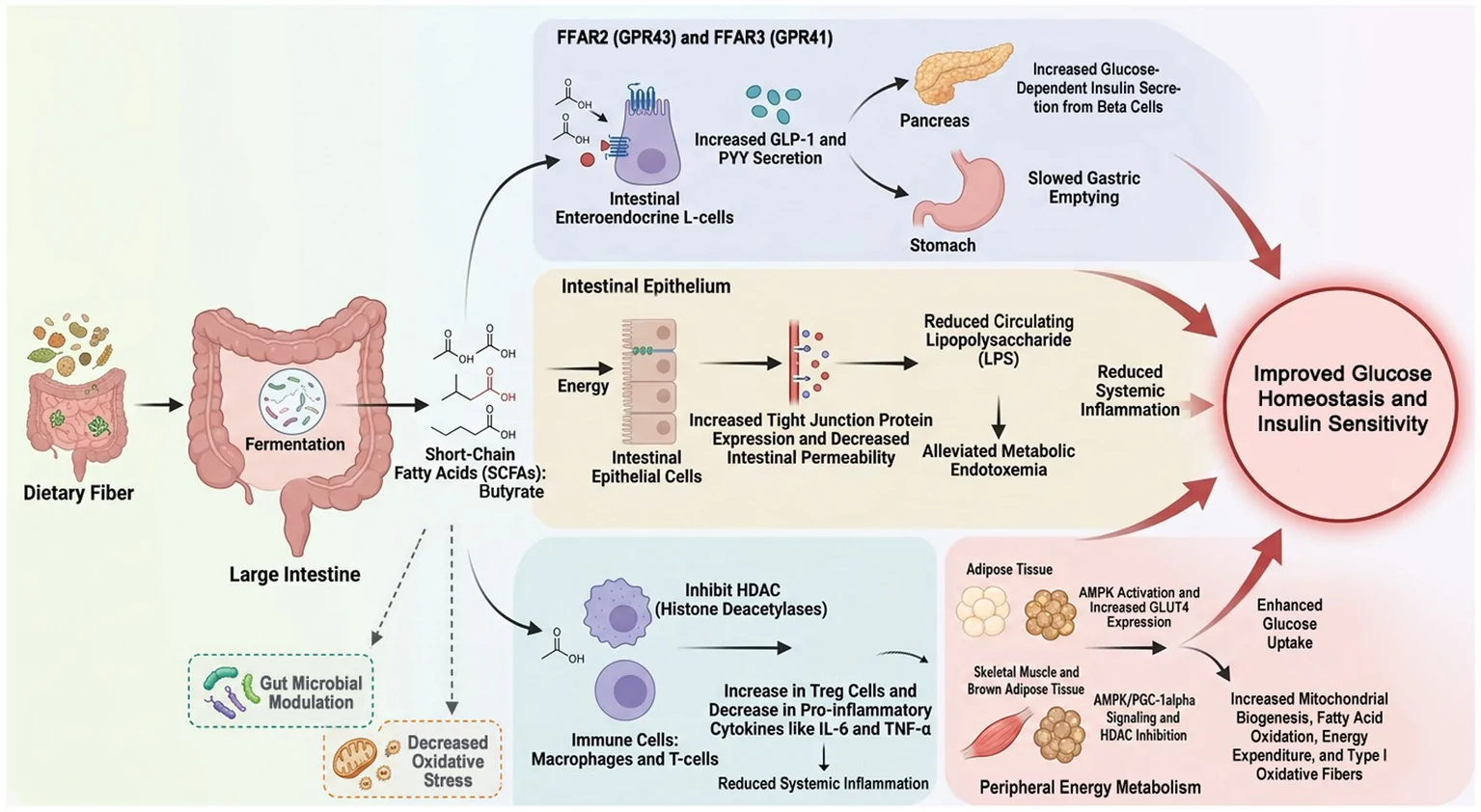
Proposed mechanisms linking butyrate to glucose homeostasis. Dietary fiber is fermented by gut microbiota into SCFAs (e.g., butyrate) in the large intestine. Butyrate may influence glucose homeostasis through four complementary pathways. First, it activates FFAR2 (GPR43) and FFAR3 (GPR41) on intestinal L cells to promote GLP-1 and PYY secretion, thereby enhancing glucose-dependent insulin secretion. Second, it strengthens intestinal barrier integrity by upregulating tight junction proteins, reducing circulating lipopolysaccharide (LPS) and systemic inflammation. Third, it inhibits histone deacetylases (HDACs), leading to increased regulatory T cells (Tregs) and decreased pro-inflammatory cytokines (TNF-α, IL-6). Fourth, it regulates AMPK-related glucose and energy metabolism in adipose tissue, skeletal muscle, and brown adipose tissue. Butyrate may also modulate gut microbial composition and attenuate oxidative stress. Collectively, these pathways may improve glucose homeostasis and insulin sensitivity in experimental models. FFAR, free fatty acid receptor; GPR, G-protein-coupled receptor; GLP-1, glucagon-like peptide-1; PYY, peptide YY; Treg, regulatory T cell; IL-6, interleukin-6; TNF-α, tumor necrosis factor-alpha; AMPK, AMP-activated protein kinase; GLUT4, glucose transporter type 4; PGC-1α, peroxisome proliferator-activated receptor-γ coactivator 1α.

From a mechanistic standpoint, butyrate acts as the primary energy source for colonic epithelial cells, maintaining physiological function while regulating gene expression and cellular processes via histone deacetylase inhibition (36–38). Its beneficial roles in glucose homeostasis have been validated in animal models: butyrate modulates energy metabolism and lowers peripheral glucose levels by activating G-protein-coupled receptors 41 and 43 (GPR41/43). Studies in GPR41/43 knockout mice further confirm that loss of this signaling impairs SCFA-induced GLP-1 secretion and exacerbates diet-induced obesity and insulin resistance (39–41). Additionally, butyrate stimulates AMPK activation and GLUT4 expression in adipose tissue, enhancing glucose uptake, glycolysis, and mitochondrial biogenesis, thus ameliorating glucose metabolic disorders (38, 42). Gao et al. (43) demonstrated that butyrate supplementation upregulates PGC-1α at transcriptional and protein stability levels via AMPK activation and histone deacetylase inhibition, thereby promoting fatty acid oxidation, mitochondrial respiration, and energy expenditure. Concurrently, butyrate increases type I oxidative fibers in skeletal muscle, mitigating diet-induced obesity and insulin resistance. Furthermore, butyrate synergistically alleviates insulin resistance by restoring gut microbial composition, enhancing intestinal epithelial tight junctions, reducing systemic low-grade inflammation, and attenuating oxidative stress (44–47). Our findings align with these well-established biological functions of butyrate and further confirm that its significant reduction occurs predominantly in overt diabetes (Figure 4).

### 4.4 Exploration of heterogeneity related to SCFAs

We further explored potential sources of heterogeneity among studies evaluating SCFA profiles across glycemic states. For example, the exclusion of Wang et al. (12) reduced heterogeneity for butyrate without materially altering the pooled effect estimate. As this study used a prospective nested case–control design that differed from those of the remaining studies, this finding suggests that differences in study design may contribute to between-study heterogeneity. The varying sensitivity of different SCFAs to study-specific factors may arise from differences in their physiological concentrations, metabolic pathways, and analytical stability. Acetate, characterized by a short half-life and rapid systemic metabolism, fluctuates markedly and is readily affected by dietary and other confounding factors (48, 49). Estimates of total SCFAs may be affected by variations in study quality or distorted by differences in the relative proportions of individual components, whereas heterogeneity in propionate and butyrate may stem from limitations in the available evidence. Taken together, these observations highlight the need to account for SCFA-specific sources of heterogeneity to improve cross-study comparability and the robustness of pooled estimates.

The observed discrepancy between fecal and circulating SCFA levels in individuals with T2DM likely reflects the distinct metabolic pools captured by each sample type (50). Fecal concentrations reflect the unabsorbed residue of colonic production and intestinal uptake, whereas circulating concentrations are further shaped by epithelial utilization, hepatic first-pass metabolism, and peripheral clearance (34, 51). Consequently, either reduced production or increased absorption may lower fecal concentrations, while downstream metabolism may attenuate the corresponding change in peripheral blood (52, 53). Paired human data showing that dietary and microbial features predict fecal SCFAs better than plasma SCFAs support this compartmental decoupling (54). Thus, lower fecal acetate and butyrate may indicate altered intestinal SCFA balance (51). The inverse association between fecal acetate and T2DM risk further supports an intestinal signal.

Reduced fecal butyrate may specifically reflect impaired microbial production, diminished fermentable-substrate availability or microbial cross-feeding, or increased epithelial uptake and local oxidation (54, 55). Increased peripheral utilization alone cannot directly reduce fecal butyrate unless accompanied by greater intestinal absorption (51). Recent metagenomic and community-modelling studies support impaired butyrate-producing capacity as a plausible contributor, whereas stable-isotope evidence shows that fecal concentrations may diverge from production flux (55–57). The underlying mechanism therefore cannot be determined from fecal measurements alone. These considerations collectively underscore that sample type should be treated as a key source of heterogeneity in future studies.

Although fecal butyrate showed an overall statistical difference, subgroup analysis revealed no genuine inter-subgroup effect. This contrast should thus be interpreted as a descriptive finding rather than evidence of effect modification. The low circulating abundance and greater analytical variability of butyrate may have reduced statistical precision (51); unequal numbers of studies and participants across subgroups may have further limited the power to detect an interaction (13, 54). No significant differences in propionate were observed across all subgroups. While marked hepatic extraction may dampen circulating differences, it cannot account for the lack of fecal changes, implying no uniform change in net luminal propionate balance across studies (58, 59). Nevertheless, a recent 4C study (12) also identified sex-specific associations between serum propionate and T2DM risk, suggesting that unstratified pooled analyses may mask potential clinical correlations.

Contrary to our expectations, no significant differences were observed across different detection methods, suggesting that current SCFA detection platforms (predominantly GC–MS) yield relatively consistent results across included observational studies. A 2025 methodological study (60) verified that six widely used extraction methods exhibited comparable performance in SCFA quantification; although extraction efficiencies differed, such variations exerted only minimal impacts on between-group effect sizes. Even so, standardized protocols covering both extraction and detection steps are still warranted (61).

### 4.5 Practical implications

This study demonstrates the impact of sample type on effect direction. Decreasing trends in fecal SCFAs (especially acetate and butyrate) are associated with elevated T2DM risk. These findings provide population-level evidence for the role of butyrate in glucose homeostasis and have important practical implications.

For nutritional and metabolic assessment, total and individual SCFAs show limited value in early pre-DM screening, while butyrate may represent a metabolic correlate of altered intestinal SCFA homeostasis. Although fecal and circulating measurements capture distinct and complementary metabolic pools, fecal SCFAs may be more informative for assessing gut microbial metabolic activity (54). Accordingly, our effect-size estimates provide a preliminary quantitative reference for developing gut microbiota-based nutritional and metabolic assessment tools (62).

For dietary intervention strategies, animal studies have shown that butyrate supplementation may alleviate high-fat diet-induced metabolic disturbances (43, 63). Plant polysaccharides, as another dietary component, have also been reported to increase SCFA production, providing evidence for dietary intervention approaches (64). Our meta-analysis provides population-level observational evidence that lower butyrate is associated with established T2DM. However, it does not demonstrate that increasing butyrate prevents T2DM or improves glycemic outcomes. Thus, the proposed nutritional cascade linking higher dietary fiber intake, increased fecal butyrate, and lower diabetes risk remains biologically plausible but clinically unconfirmed (30, 65, 66). Butyrate-boosting dietary strategies and supplementation therefore warrant evaluation in randomized controlled trials before they can be recommended as adjunctive interventions for diabetes management.

### 4.6 Strengths and limitations

This study has several key strengths. It includes both early and recent publications, integrating historical evidence with up-to-date findings. We investigated the dynamic associations of individual SCFAs across glycemic status, evaluated the influences of sample type and detection method, and explored the underlying mechanisms of key SCFA in diabetes pathogenesis.

Nevertheless, several limitations should be acknowledged. (1) Given the wide variety of SCFAs and multiple confounding factors including diet and BMI, comprehensive stratification and subgroup analyses were not feasible. Meanwhile, prominent inherent heterogeneity in gut microbial metabolites further limited the interpretation of our findings; (2) Stratification by SCFA type and glycemic status resulted in a limited numbers of studies for each metabolite, with some only eligible for qualitative synthesis; comprehensive subgroup analyses and publication bias assessments were not always feasible, which limited the robustness and generalizability of our findings; (3) The limited number of prospective cohort studies and the absence of dose–response analyses resulted in relatively weak evidence for causal inference.

## 5 Conclusion

SCFA alterations were more evident in established T2DM than in pre-DM, with reduced butyrate emerging as the most consistent metabolic finding. Acetate exhibited variable patterns across the glycemic comparisons and biological matrices. Further long-term prospective cohort studies are warranted to determine whether specific SCFA alterations precede T2DM and have value for early identification, while mechanistic and interventional studies are needed to clarify the underlying mechanisms and potential therapeutic relevance.

## Data Availability

The original contributions presented in the study are included in the article/Supplementary material, further inquiries can be directed to the corresponding authors.
