# Supplementary material for: Changes in short-chain fatty acids across the glycemic spectrum: a systematic review and meta-analysis
Source: Front Public Health. 2026 Sep 11;14:1912172. doi: 10.3389/fpubh.2026.1912172 (PMC13612369; doi:10.3389/fpubh.2026.1912172)
Supplement: Supplementary file 1 [file data_sheet_1.docx]

Supplementary Figure 1 Forest plots. (A) Butyrate levels between T2DM and NGT; (B) Valerate levels between T2DM and NGT.

Supplementary Figure 2 Meta-analysis for the comparison of continuous outcome measures (namely acetate, propionate, caproate and total SCFAs) between T2DM and NGT.

Supplementary Figure 3 Meta-analysis of binary outcome measures (namely acetate, butyrate, and total SCFAs) for T2DM risk.

Supplementary Figure 4 Meta-analysis of binary outcome measures (namely propionate, valerate, and caproate) for T2DM risk.

Supplementary Figure 5 Meta-analysis for the comparison of continuous outcome measures (namely acetate, propionate and butyrate) between pre-DM and NGT.

Supplementary Figure 6 Meta-analysis of dichotomous outcome (acetate) on the risk of incident pre-DM.

Supplementary Figure 7 Meta-analysis for the comparison of continuous outcome measure (propionate) between T2DM and pre-DM.

Supplementary Figure 8 Subgroup analysis by diabetes stage and acetate (Sample type).

Supplementary Figure 9 Subgroup analysis by diabetes stage and acetate (Testing methods).

Supplementary Figure 10 Subgroup analysis by diabetes stage and propionate (Sample type).

Supplementary Figure 11 Subgroup analysis by diabetes stage and butyrate (Sample type).

Supplementary Figure 12 Publication bias in T2DM vs NGT (continuous/dichotomous acetate).

Supplementary Figure 13 Publication bias in T2DM vs NGT (continuous propionate).

Supplementary Figure 14 Publication bias in T2DM vs NGT (continuous butyrate).
