# Supplementary material for: Changes in short-chain fatty acids across the glycemic spectrum: a systematic review and meta-analysis
Source: Front Public Health. 2026 Sep 11;14:1912172. doi: 10.3389/fpubh.2026.1912172 (PMC13612369; doi:10.3389/fpubh.2026.1912172)
Supplement: Supplementary file 2 [file table_1.docx]

Table S1 Search algorithms for each database

| **Database** | **Search Algorithm** |
| --- | --- |
| PubMed | (((((impaired fasting glucose[Title/Abstract]) OR ((Glucose Intolerance[MeSH Terms]) OR (((Glucose Intolerances[Title/Abstract]) OR (Impaired Glucose Tolerance[Title/Abstract])) OR (Impaired Glucose Tolerances[Title/Abstract])))) OR ((Prediabetic State[MeSH Terms]) OR ((Prediabetic States[Title/Abstract]) OR (Prediabetes[Title/Abstract])))) OR ((Diabetes Mellitus, Type 2[MeSH Terms]) OR ((((((((((((((T2DM[Title/Abstract]) OR (Type 2 Diabetes Mellitus[Title/Abstract])) OR (Type 2 Diabetes[Title/Abstract])) OR (Diabetes Mellitus, Type II[Title/Abstract])) OR (Stable Diabetes Mellitus[Title/Abstract])) OR (Adult-Onset Diabetes Mellitus[Title/Abstract])) OR (Ketosis-Resistant Diabetes Mellitus[Title/Abstract])) OR (Non-Insulin-Dependent Diabetes Mellitus[Title/Abstract])) OR (Noninsulin-Dependent Diabetes Mellitus[Title/Abstract])) OR (NIDDM[Title/Abstract])) OR (Maturity-Onset Diabetes Mellitus[Title/Abstract])) OR (Maturity-Onset Diabetes[Title/Abstract])) OR (MODY[Title/Abstract])) OR (Slow-Onset Diabetes Mellitus[Title/Abstract])))) OR ((Insulin Resistance[MeSH Terms]) OR (Insulin Sensitivity[Title/Abstract]))) AND (((((Fatty Acids, Volatile[MeSH Terms]) OR ((((Volatile Fatty Acids[Title/Abstract]) OR (Short-Chain Fatty Acids[Title/Abstract])) OR (Short-Chain Fatty Acid[Title/Abstract])) OR (Volatile Fatty Acid[Title/Abstract]))) OR ((Butyrates[MeSH Terms]) OR ((((n-Butyrate[Title/Abstract]) OR (Butyrate[Title/Abstract])) OR (Butyric Acids[Title/Abstract])) OR (Butanoic Acids[Title/Abstract])))) OR ((Propionates[MeSH Terms]) OR (((((((Propionate[Title/Abstract]) OR (Propanoate[Title/Abstract])) OR (Propanoates[Title/Abstract])) OR (Propionic Acids[Title/Abstract])) OR (Propionic Acid Derivatives[Title/Abstract])) OR (Propanoic Acid Derivatives[Title/Abstract])) OR (Propanoic Acids[Title/Abstract])))) OR ((Acetates[MeSH Terms]) OR (((Acetate[Title/Abstract]) OR (Acetic Acid Esters[Title/Abstract])) OR (Acetic Acids[Title/Abstract])))) |
| Web of science | ((“impaired fasting glucose” OR “Glucose Intolerance” OR “Glucose Intolerances” OR “Impaired Glucose Tolerance” OR “Impaired Glucose Tolerances” OR “Prediabetic State” OR “Prediabetic States” OR “Prediabetes” OR “Diabetes Mellitus, Type 2” OR “T2DM” OR “Type 2 Diabetes Mellitus” OR “Type 2 Diabetes” OR “Diabetes Mellitus, Type II” OR “Stable Diabetes Mellitus” OR “Adult-Onset Diabetes Mellitus” OR “Ketosis-Resistant Diabetes Mellitus” OR “Non-Insulin-Dependent Diabetes Mellitus” OR “Noninsulin-Dependent Diabetes Mellitus” OR “NIDDM” OR “Maturity-Onset Diabetes Mellitus” OR “Maturity-Onset Diabetes” OR “MODY” OR “Slow-Onset Diabetes Mellitus” OR “Insulin Resistance” OR “Insulin Sensitivity”) AND (“Fatty Acids, Volatile” OR “Volatile Fatty Acids” OR “Short-Chain Fatty Acids” OR “Short-Chain Fatty Acid” OR “Volatile Fatty Acid” OR “Butyrates” OR “n-Butyrate” OR “Butyrate” OR “Butyric Acids” OR “Butanoic Acids” OR “Propionates” OR “Propionate” OR “Propanoate” OR “Propanoates” OR “Propionic Acids” OR “Propionic Acid Derivatives” OR “Propanoic Acid Derivatives” OR “Propanoic Acids” OR “Acetates” OR “Acetate” OR “Acetic Acid Esters” OR “Acetic Acids”)) |
| Cochrane Library | #1 (impaired fasting glucose):ti,ab,kw  #2 MeSH descriptor: [Glucose Intolerance] explode all trees  #3 (Glucose Intolerances):ti,ab,kw  #4 (Impaired Glucose Tolerance):ti,ab,kw  #5 (Impaired Glucose Tolerances):ti,ab,kw  #6 #2 OR #3 OR #4 OR #5  #7 MeSH descriptor: [Prediabetic State] explode all trees  #8 (Prediabetic States):ti,ab,kw  #9 (Prediabetes):ti,ab,kw  #10 #7 OR #8 OR #9  #11 MeSH descriptor: [Diabetes Mellitus, Type 2] explode all trees 27561  #12 (T2DM):ti,ab,kw 10050  #13 (Type 2 Diabetes Mellitus):ti,ab,kw 59287  #14 (Type 2 Diabetes):ti,ab,kw 68072  #15 (Diabetes Mellitus, Type II):ti,ab,kw 5818  #16 (Stable Diabetes Mellitus):ti,ab,kw 4643  #17 (Adult-Onset Diabetes Mellitus):ti,ab,kw 102  #18 (Ketosis-Resistant Diabetes Mellitus):ti,ab,kw 2  #19 (Non-Insulin-Dependent Diabetes Mellitus):ti,ab,kw 23917  #20 (Noninsulin-Dependent Diabetes Mellitus):ti,ab,kw 558  #21 (NIDDM):ti,ab,kw 1137  #22 (Maturity-Onset Diabetes Mellitus):ti,ab,kw 91  #23 (Maturity-Onset Diabetes):ti,ab,kw 103  #24 (MODY):ti,ab,kw 46  #25 (Slow-Onset Diabetes Mellitus):ti,ab,kw 4  #26 #11 OR #12 OR #13 OR #14 OR #15 OR #16 OR #17 OR #18 OR #19 OR #20 OR #21 OR #22 OR #23 OR #24 OR #25 72314  #27 MeSH descriptor: [Insulin Resistance] explode all trees 9921  #28 (Insulin Sensitivity):ti,ab,kw 12159  #29 #27 OR #28 18375  #30 MeSH descriptor: [Fatty Acids, Volatile] explode all trees 5202  #31 (Volatile Fatty Acids):ti,ab,kw 444  #32 (Short-Chain Fatty Acids):ti,ab,kw 1553  #33 (Short-Chain Fatty Acid):ti,ab,kw 1092  #34 (Volatile Fatty Acid):ti,ab,kw 267  #35 #30 OR #31 OR #32 OR #33 OR #34 6862  #36 MeSH descriptor: [Butyrates] explode all trees 6113  #37 (n-Butyrate):ti,ab,kw 8  #38 (Butyrate):ti,ab,kw 1289  #39 (Butyric Acids):ti,ab,kw 217  #40 (Butanoic Acids):ti,ab,kw 3  #41 #36 OR #37 OR #38 OR #39 OR #40 7351  #42 MeSH descriptor: [Propionates] explode all trees 3496  #43 (Propionate):ti,ab,kw 4551  #44 (Propanoate):ti,ab,kw 9  #45 (Propanoates):ti,ab,kw 0  #46 (Propionic Acids):ti,ab,kw 148  #47 (Propionic Acid Derivatives):ti,ab,kw 23  #48 (Propanoic Acid Derivatives):ti,ab,kw 5  #49 (Propanoic Acids):ti,ab,kw 8  #50 #42 OR #43 OR #44 OR #45 OR #46 OR #47 OR #48 OR #49 8130  #51 MeSH descriptor: [Acetals] explode all trees 11  #52 (Acetate):ti,ab,kw 13205  #53 (Acetic Acid Esters):ti,ab,kw 15  #54 (Acetic Acids):ti,ab,kw  #55 #51 OR #52 OR #53 OR #54  #56 #1 OR #6 OR #10 OR #26 OR #29  #57 #35 OR #41 OR #50 OR #55  #58 #56 AND #57 |
| Embase | #1 'insulin resistance'/exp OR 'insulin sensitivity':ab,ti OR 'impaired fasting glucose':ab,ti OR 'glucose intolerance'/exp OR 'glucose intolerances':ab,ti OR 'impaired glucose tolerance':ab,ti OR 'impaired glucose tolerances':ab,ti OR 'prediabetic state'/exp OR 'prediabetic states':ab,ti OR prediabetes:ab,ti OR 'diabetes mellitus, type 2'/exp OR t2dm:ab,ti OR 'type 2 diabetes mellitus':ab,ti OR 'type 2 diabetes':ab,ti OR 'diabetes mellitus, type ii':ab,ti OR 'stable diabetes mellitus':ab,ti OR 'adult-onset diabetes mellitus':ab,ti OR 'ketosis-resistant diabetes mellitus':ab,ti OR 'non-insulin-dependent diabetes mellitus':ab,ti OR 'noninsulin-dependent diabetes mellitus':ab,ti OR niddm:ab,ti OR 'maturity-onset diabetes mellitus':ab,ti OR 'maturity-onset diabetes':ab,ti OR mody:ab,ti OR 'slow-onset diabetes mellitus':ab,ti  #2 'fatty acids, volatile'/exp OR 'volatile fatty acids':ab,ti OR 'short-chain fatty acids':ab,ti OR 'short-chain fatty acid':ab,ti OR 'volatile fatty acid':ab,ti OR 'butyrates'/exp OR 'n butyrate':ab,ti OR butyrate:ab,ti OR 'butyric acids':ab,ti OR 'butanoic acids':ab,ti OR 'propionates'/exp OR propionate:ab,ti OR propanoate:ab,ti OR propanoates:ab,ti OR 'propionic acids':ab,ti OR 'propionic acid derivatives':ab,ti OR 'propanoic acid derivatives':ab,ti OR 'propanoic acids':ab,ti OR 'acetates'/exp OR acetate:ab,ti OR 'acetic acid esters':ab,ti OR 'acetic acids':ab,ti  #1 AND #2 |

Table S2 Agency for Health Care Research and Quality (AHRQ) quality assessment summary

| **Author, year** | **Definition** | **Sample selection** | **Time period** | **Consecution** | **Subjective components** | **Assessments for quality assurance purposes** | **Any patient exclusions from analysis** | **Confounding** | **Missing data** | **Response rates and completeness** | **Follow-up** | **Scores/11** | **Quality** |
| --- | --- | --- | --- | --- | --- | --- | --- | --- | --- | --- | --- | --- | --- |
| Akanji 1988 | 1 | 0 | 0 | 0 | 0.5 | 1 | 0.5 | 0 | 0.5 | 0 | 0.5 | 4 | medium quality |
| Akanji 1989 | 1 | 0 | 0 | 1 | 0.5 | 1 | 0.5 | 1 | 0.5 | 0 | 0.5 | 6 | high quality |
| Wolever 1997 | 1 | 1 | 0 | 0 | 0.5 | 1 | 0.5 | 1 | 0.5 | 0 | 0 | 5.5 | medium quality |
| Liu 2016 | 1 | 1 | 1 | 1 | 0.5 | 1 | 0.5 | 1 | 0.5 | 0 | 0.5 | 8 | high quality |
| Morais 2021 | 1 | 1 | 0 | 0.5 | 0.5 | 1 | 0.5 | 1 | 0.5 | 0 | 0.5 | 6.5 | high quality |
| Zhong 2021 | 1 | 1 | 1 | 0.5 | 0.5 | 1 | 1 | 1 | 1 | 0 | 0.5 | 8.5 | high quality |
| Salamone 2022 | 1 | 0.5 | 0 | 0.5 | 0.5 | 0.5 | 0.5 | 1 | 0.5 | 0 | 0.5 | 5.5 | medium quality |
| Smith 2023 | 1 | 1 | 0 | 0.5 | 0.5 | 1 | 1 | 1 | 0.5 | 0 | 0.5 | 7 | high quality |
| Liu 2024 | 1 | 1 | 0 | 0.5 | 0.5 | 1 | 1 | 1 | 1 | 0 | 0.5 | 7.5 | high quality |
| Shinoda 2024 | 1 | 1 | 0 | 0.5 | 0.5 | 1 | 0.5 | 1 | 0.5 | 0 | 0.5 | 6.5 | high quality |
| Zhang 2024 | 1 | 1 | 1 | 0.5 | 0.5 | 1 | 0.5 | 1 | 0.5 | 0 | 0.5 | 7.5 | high quality |
| Puig 2025 | 1 | 0 | 0 | 0.5 | 0.5 | 1 | 0.5 | 1 | 1 | 0 | 0.5 | 6 | high quality |

Table S3 Newcastle-Ottawa Scale (NOS) quality assessment summary

| **Author, year** | **Selection** | | | | **Comparability** | **Outcome** | | | **Scores/9** | **Quality** |
| --- | --- | --- | --- | --- | --- | --- | --- | --- | --- | --- |
|  | **Representativeness**  **of the exposed cohort** | **Selection of the non exposed cohort** | **Ascertainment**  **of exposure** | **Demonstration that outcome of interest was not present at start of study** | **Comparability** | **Assessment**  **of outcome** | **Follow-up** | **Adequacy of**  **follow up of cohorts** |  |  |
| Adachi 2019 |  | ★ | ★ | ★ | ★★ | ★ |  | ★ | 7 | medium quality |
| Li 2024 | ★ | ★ | ★ | ★ | ★★ | ★ | ★ |  | 8 | high quality |
| Yang 2024 | ★ | ★ | ★ | ★ | ★★ | ★ | ★ |  | 8 | high quality |
| Llauradó 2025 | ★ | ★ | ★ | ★ | ★★ | ★ | ★ |  | 8 | high quality |
| Wang 2025 | ★ | ★ | ★ | ★ | ★★ | ★ | ★ | ★ | 9 | high quality |

Table S4 Association analysis between T2D stage and SCFAs

| **Comparison groups** | **SCFA type** | **Variable types** | **Included studies** | **Pooled effect size (95% CI)** | ***I²*** | ***P*** |
| --- | --- | --- | --- | --- | --- | --- |
| T2D vs. NGT | Acetate | SMD | 9 (n=4030) | -0.09 [-0.38, 0.20] | 81 %, *P*<0.00001 | 0.55 |
|  |  | OR | 5 (n=3753) | 0.90 [0.74, 1.08] | 85 %, *P*<0.0001 | 0.25 |
|  | Propionate | SMD | 6 (n=6320) | -0.04 [-0.25, 0.18] | 85 %, *P*<0.00001 | 0.74 |
|  |  | OR | 2 (n=3514) | 1.04 [0.97, 1.11] | 0 %, *P*=0.48 | 0.27 |
|  | Butyrate | SMD | 6 (n=3787) | -0.49 [-0.96, -0.01] | 90 %, *P*<0.00001 | 0.04 |
|  |  | OR | 4 (n=6247) | 0.99 [0.86, 1.15] | 77 %, *P*=0.005 | 0.94 |
|  | Valerate | SMD | 4 (n=3616) | 0.07 [0.01, 0.14] | 0 %, *P*=0.66 | 0.03 |
|  |  | OR | 2 (n=3514) | 1.06 [0.98, 1.14] | 33 %, *P*=0.22 | 0.15 |
|  | Caproate | SMD | 2 (n=3434) | 0.06 [-0.01, 0.13] | 39 %, *P*=0.20 | 0.09 |
|  |  | OR | 2 (n=3514) | 1.06 [0.98, 1.14] | 0 %, *P*=0.32 | 0.14 |
|  | Total SCFAs | SMD | 3 (n=3603) | -0.41 [-0.97, 0.16] | 90 %, *P*<0.0001 | 0.16 |
|  |  | OR | 3 (n=3580) | 0.74 [0.44, 1.25] | 84 %, *P*=0.002 | 0.27 |
| Pre-DM vs. NGT | Acetate | SMD | 4 (n=141) | -0.09 [-0.43, 0.25] | 17 %, *P*=0.31 | 0.61 |
|  |  | OR | 2 (n=133) | 1.18 [0.90, 1.55] | 71 %, *P*=0.06 | 0.23 |
|  | Propionate | SMD | 4 (n=2373) | 0.24 [-0.24, 0.72] | 75 %, *P*=0.007 | 0.33 |
|  |  | OR | 1 (n=63) | 0.71 [0.53, 0.96] | NR | 0.02 |
|  | Butyrate | SMD | 3 (n=132) | 0.24 [-0.42, 0.90] | 68 %, *P*=0.04 | 0.47 |
|  | Valerate | SMD | 1 (n=63) | -0.17 [-0.68, 0.34] | NR | 0.51 |
|  | Total SCFAs | SMD | 1 (n=29) | -0.20 [-0.99, 0.59] | NR | 0.61 |
| T2D vs. pre-DM | Acetate | SMD | 1 (n=49) | -0.71 [-1.29, -0.13] | NR | 0.02 |
|  |  | OR | 1 (n=49) | 0.56 [0.37, 0.85] | NR | 0.006 |
|  | Propionate | SMD | 2 (n=975) | 0.03 [-0.11, 0.17] | 0 %, *P*=0.33 | 0.66 |
|  | Butyrate | SMD | 1 (n=49) | -0.50 [-1.07, 0.07] | NR | 0.09 |
|  | Valerate | SMD | 1 (n=49) | 0.17 [-0.39, 0.73] | NR | 0.55 |

Three comparisons were performed: T2D vs. NGT, Pre-DM vs. NGT, and T2D vs. Pre-DM. Effect sizes are presented as SMD or OR, with 95% CI. The number of included studies and total sample size (n) are shown. Heterogeneity was evaluated using the *I²* statistic, and statistical significance was set at *P* < 0.05. Abbreviations: T2D, type 2 diabetes; Pre-DM, prediabetes; NGT, normal glucose tolerance; OR, odds ratio; SMD: standardized mean difference; CI: confidence intervals; NR, Not Reported.

Table S5 Subgroup analyses of SCFAs in the comparison of T2D versus NGT

| **Acetate Subgroups** | **Included studies** | **SMD (95% CI)** | ***I^2^* (%)** | ***P*-values** | ***P*-value between groups** |
| --- | --- | --- | --- | --- | --- |
| **Sample type** | | | | | |
| Fecal SCFAs | 3 (n=282) | -0.46 [-0.70, -0.22] | 0 | 0.0002 | <0.05 |
| Circulating SCFAs | 6 (n=3748) | 0.14 [-0.24, 0.53] | 81 | 0.47 |  |
| **Testing methods** | | | | | |
| Chromatography | 6 (n=3787) | -0.28 [-0.61, 0.04] | 78 | 0.08 | >0.05 |
| Other testing methods | 3 (n=243) | 0.40 [-0.31, 1.12] | 74 | 0.27 |  |
| **Acetate Subgroups** | **Included studies** | **OR (95% CI)** | ***I^2^* (%)** | ***P*-values** | ***P*-value between groups** |
| **Sample type** | | | | | |
| Fecal SCFAs | 3 (n=266) | 0.23 [0.09, 0.57] | 52 | 0.001 | <0.05 |
| Circulating SCFAs | 2 (n=3487) | 1.02 [0.99, 1.06] | 3 | 0.17 |  |
| **Testing methods** | | | | | |
| Chromatography | 3 (n=3614) | 0.29 [0.05, 1.58] | 89 | 0.15 | >0.05 |
| Other testing methods | 2 (n=139) | 0.69 [0.29, 1.63] | 87 | 0.40 |  |
| **Propionate Subgroups** | **Included studies** | **SMD (95% CI)** | ***I^2^* (%)** | ***P*-values** | ***P*-value between groups** |
| **Sample type** | | | | | |
| Fecal SCFAs | 2 (n=182) | -0.02 [-0.81, 0.76] | 84 | 0.95 | >0.05 |
| Circulating SCFAs | 4 (n=6138) | -0.05 [-0.30, 0.19] | 89 | 0.67 |  |
| **Butyrate Subgroups** | **Included studies** | **SMD (95% CI)** | ***I^2^* (%)** | ***P*-values** | ***P*-value between groups** |
| **Sample type** | | | | | |
| Fecal SCFAs | 3 (n=282) | -0.58 [-0.91, -0.25] | 46 | 0.0006 | >0.05 |
| Circulating SCFAs | 3 (n=3505) | -0.41 [-1.30, 0.48] | 91 | 0.37 |  |

Subgroup analyses were performed for acetate, propionate, and butyrate in the comparison of T2D vs. NGT. For acetate, subgroup analyses were conducted by sample type (fecal vs. circulating SCFAs) and testing methods (chromatography vs. other methods). Effect sizes were presented as SMD or OR, each with corresponding 95% CI. Due to the limited number of included studies, subgroup analyses by testing method and OR-based analyses were not performed for propionate and butyrate. The number of included studies and total sample size (n) are shown. Between-study heterogeneity was assessed using the *I²* statistic, and *P*-values for within-subgroup and between-subgroup differences are reported. Statistical significance was defined as *P* < 0.05. Abbreviations: T2D, type 2 diabetes; NGT, normal glucose tolerance; OR, odds ratio; SMD: standardized mean difference; CI: confidence intervals.
